# Novel Design in Venturi-Type Nozzle by Selective Laser Melting for Enhancement in Microbubble Generation

**DOI:** 10.3390/mi17050547

**Published:** 2026-04-29

**Authors:** Minhoo Chung, Changkyoo Park

**Affiliations:** Department of Materials Science and Engineering, Seoul National University of Science and Technology, Seoul 01811, Republic of Korea

**Keywords:** Venturi nozzle, selective laser melting, microbubble, cavitation

## Abstract

This study applies selective laser melting (SLM) to fabricate stainless steel 316L (SS316L) structures on the distribution plate of a Venturi-type nozzle in a pressurized dissolution microbubble generator. SLM is employed because the fabricated structures are approximately hundreds of micrometers in size, making them difficult to produce using conventional milling or other machining methods. These structures are designed to enhance cavitation and gas–liquid interaction, thereby enhancing microbubble generation. Various conditions of the SLM process are conducted, and the combination of 140 W laser power, 100 mm/s scan speed, 30 µm layer thickness, and 120 µm hatch distance achieves the highest relative density while maintaining the austenite phase of SS316L, thus being selected as the optimal SLM process parameters. Microbubble generation test are conducted under three different dissolution tank pressure conditions (0.20, 0.25, and 0.30 MPa) using nozzles with and without the SLM structures. The generated microbubbles in both nozzles ranges from 1 to 110 µm, satisfying the size conditions for microbubbles. The average microbubble size is smaller in the SLM-assisted nozzle (31.8 µm) compared with the plain nozzle (38.8 µm). Furthermore, under the dissolution tank pressure of 0.30 MPa for 30 s, the SLM-assisted nozzle generates a maximum of 52,368 microbubbles, representing approximately a 102.1% increase compared with the plain nozzle (25,907 microbubbles). These results demonstrate that incorporating SLM structures to Venturi-type nozzle effectively enhances microbubble generation, offering promising potential for applications in water treatment, biomedical processes, and chemical engineering.

## 1. Introduction

Additive manufacturing (AM), commonly known as 3D printing, is a layer-by-layer fabrication technology, in which powder materials are sequentially deposited to construct three-dimensional structures. Compared with conventional manufacturing processes such as subtractive machining or casting, AM enables the fabrication of highly complex geometries and structures that are difficult to realize using traditional techniques. Consequently, AM has attracted significant attention as a key manufacturing technology in the era of the Fourth Industrial Revolution [1,2].

Although AM technology has been developed for more than two decades, its early applications were limited by material constraints and the insufficient maturity of printing technologies. As a result, it was mainly used for fabricating porous structures or rapid prototypes during the early stages of product development [3]. However, continuous advancements in materials and process control have significantly improved the density and mechanical properties of additively manufactured components. Today, AM is widely applied in various industries, including medical devices, aircraft engine components [4,5], and automotive parts [6]. AM technology fabricates structures directly from user-defined CAD data, offering high design flexibility and enabling the production of small and geometrically complex components [7,8]. Among the various AM techniques, selective laser melting (SLM) is a representative powder bed fusion process that enables the fabrication of complex metallic components with high precision. Owing to these advantages, SLM has gained considerable attention across multiple industrial fields [9].

Microbubbles are defined as gas bubbles with diameters smaller than 100 μm, exhibiting a large specific surface area [10]. Due to their small buoyancy, microbubbles rise slowly in liquid. In addition, the gas–liquid interface of microbubbles possesses a negative surface charge, enabling the adsorption of fine particles. Furthermore, the collapse of microbubbles can generate free radicals, which contribute to their sterilization capability [11]. Owing to these characteristics, microbubble technology has recently attracted increasing attention in various fields such as agriculture, wastewater treatment, food processing, medical applications, and semiconductor manufacturing. Accordingly, extensive research has been conducted on both the functional properties of microbubbles and the development of microbubble generation technologies [12,13]. In particular, improving the efficiency of pollutant removal using microbubbles has become one of the most actively studied research topics [14]. The high pollutant removal capability of microbubbles can enhance the energy efficiency of wastewater treatment processes and improve water purification, which in turn supports plant growth and agricultural productivity [15].

Microbubble generators can be classified according to their operating principles into dissolved air flotation [16], static mixer [17], rotary liquid flow [18], and nozzle-based systems [19,20]. In most systems except the nozzle-based system, a two-phase flow, composed of air and water, must firstly generates before an occurrence of bubble fragmentation. However, this process introduces significant flow resistance, resulting in large pressure losses and reduced energy efficiency. In addition, rotating mechanical components are typically required to fragment bubbles, which leads to complex structures and increased maintenance requirements [16,21]. In contrast, nozzle-type systems can generate a static pressure lower than atmospheric pressure depending on the nozzle geometry, allowing air to be automatically entrained into the liquid flow. Among them, Venturi-type nozzles are widely used due to their relatively small pressure drop and low electric power consumption for air induction. Moreover, the absence of mechanically moving components provides advantages in terms of structural simplicity and energy efficiency. Nevertheless, conventional Venturi nozzles have limitations in generating high-density microbubbles because they cannot sufficiently fragment air bubbles [22]. In addition, the generated bubbles tend to be relatively large (approximately a few hundred micrometers) compared with those produced by other microbubble generation methods, indicating the need for additional design strategies to an increase the number of microbubble and a reduction in bubble size [23,24,25].

A typical Venturi nozzle consists of a throat section where the cross-sectional area first decreases and then expands. As the fluid passes through the narrow throat section, the pressure decreases, which facilitates microbubble generation. Recently, alternative approaches based on impinging jet flow have attracted attention for enhancing bubble generation performance. The impinging jet system is consisted of the distribution plate attached to the Venturi-type nozzle, and a liquid jet from the nozzle collides with a fixed distribution plate, creating a region where is highly concentrated turbulence intensity. This mechanism can effectively reduce bubble size while maintaining high energy efficiency [26]. Zhu et al. investigated the bubble growth and detachment mechanisms in such impinging jet systems through both experimental and numerical analyses [27]. However, the number of generated microbubbles is still low with limited distribution distance of generated microbubbles.

To maximize the number of microbubble generation in a Venturi nozzle, this study proposed a hybrid strategy that combined an introduction of the distribution plate and SLM-built structures. To realize this concept, the SLM process was adopted to fabricate the multiple sub-millimeter-sized of the SLM structures on the distribution plate. First, identifying the optimal SLM process was conducted, then this condition of SLM process were used to manufacture the SLM structures on the distribution plate. When the fluid discharged from the nozzle and impinged on both the plate and the SLM-fabricated structure, vortex generation was intensified. Furthermore, the flow disturbance induced by the SLM structure locally generated low-pressure regions within the fluid, promoting cavitation and thereby facilitating microbubble formation. The microbubble generation performance was experimentally compared before and after the introduction of the SLM structure to the Venturi-type nozzle, and a computational fluid dynamics (CFD) simulation was also performed to elucidate the mechanism of microbubble generation.

## 2. Experimental Setup

### 2.1. Venturi-Type Nozzle for Microbubble Generation

The mechanism of microbubble generation in a Venturi nozzle is governed by Bernoulli’s principle and Henry’s law. When water is pressurized by a pump, the its pressure value increases. The increased pressure enhances the solubility of oxygen in water according to Henry’s law, which states that the solubility of a gas in a liquid is proportional to its partial pressure. Consequently, a greater amount of oxygen gas can dissolve in water under high-pressure conditions [28]. As the pressurized water flows through the Venturi nozzle, it passes through the narrow throat section. In this section, the water flow velocity increases, while the gas pressure decreases [29]. This relationship follows Henry’s law and Bernoulli’s principle, as described in Equations (1) and (2).

(1) $$C=H\times P_{g}$$

Here, *C* represents the solubility of a gas in a given solvent at a specific temperature, *H* is Henry’s law constant, and *P_g_* denotes the partial pressure of the gas. Therefore, when the liquid undergoes depressurization, the solubility of the dissolved gas decreases, resulting in the release of gas molecules from the liquid phase [28].

(2) $$\mathrm{Bernoulli}’s\;\mathrm{equation}=P+\frac{pv^{2}}{2}+\rho gh=\mathrm{CONSTANT}$$

**Figure 1:**
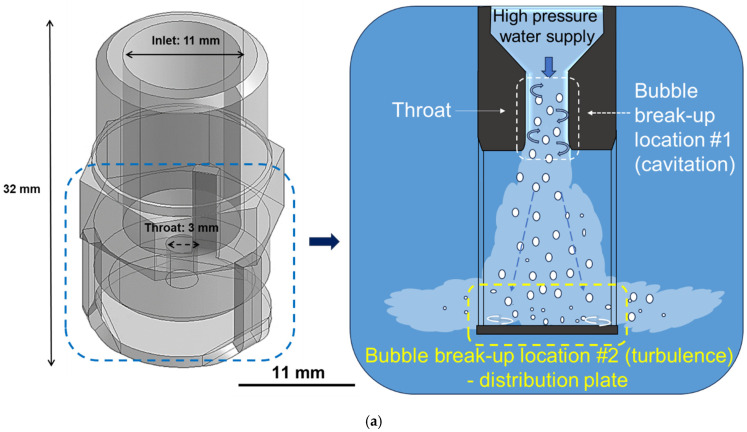

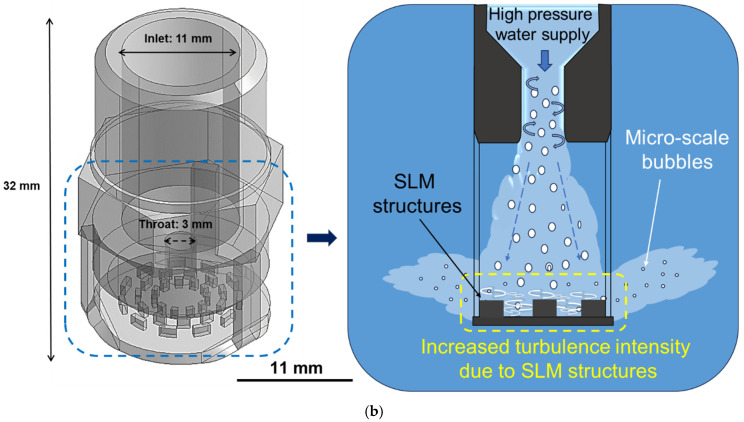
Different nozzle types for the microbubble generation. (**a**) Venturi-type nozzle with the distribution plate and (**b**) Venturi-type nozzle with the distribution plate and SLM structures.

In Bernoulli’s equation, P represents the static pressure of the liquid, p is the density of the fluid, v is the flow velocity, g is the gravitational acceleration, and h is the elevation height of the fluid. When oxygen dissolved in water passes through the Venturi nozzle, which has a constricted cross-sectional area (i.e., throat), and is subsequently depressurized rapidly to atmospheric pressure, the velocity of the fluid increases while the static pressure decreases at the throat section owing to a spatial dimension change. This pressure reduction can induce cavitation, resulting in the primary formation of bubbles. Furthermore, when the fluid exits the nozzle and experiences rapid depressurization to atmospheric pressure, the dissolved oxygen is released from the liquid and separates as gas bubbles. At this stage, fine bubbles, known as microbubbles, are formed, as illustrated in Figure 1a [30].

In this study, a customized Venturi-type nozzle is designed to generate microbubbles under a maximum flow rate condition of 30 L/min. The microbubble generation mechanism and the schematic illustration of the customized Venturi nozzle are shown in Figure 1a. The total length of the nozzle is 32 mm. The nozzle inlet has dimensions of 11 (D) × 19 (L) mm^2^, which gradually decreases to 3 (D) × 4 (L) mm^2^ at the throat section, where microbubbles were generated through cavitation. Cavitation refers to the phenomenon in which small vapor bubbles develop, when the static pressure of a liquid becomes lower than its vapor pressure [31]. Such a reduction in static pressure can occur when the cross-sectional area of the fluid flow decreases within a Venturi nozzle, as described earlier.

Figure 1a shows the customized Venturi nozzle with the distribution plate. In this study, as shown in Figure 1a, the distribution plate was positioned below the nozzle, and the center of the throat and the center of the distribution plate were precisely aligned. Microbubbles generated by cavitation at the throat section were discharged from the nozzle in a direction perpendicular to the liquid jet flow. When the liquid jet collided with a plate, the direction of the liquid flow changed from vertical to radial. As a result, the microbubbles were dispersed radially into the surrounding region. This mechanism enabled the formation of a more uniform distribution of microbubbles within the liquid. Furthermore, the collision of the liquid jet with the distribution plate generated strong turbulence, which promoted the fragmentation of microbubbles. Consequently, the bubble size can be reduced while the number of bubbles increases [26,27]. In this study, as shown in Figure 1a, the distribution plate was positioned below the nozzle, and the center of the throat and the center of the distribution plate were precisely aligned.

Figure 1b illustrates the Venturi nozzle incorporating both the distribution plate and an SLM-fabricated structure. A millimeter-scale structure was fabricated on the distribution plate using the selective laser melting (SLM) process. This structure was designed to enhance turbulence and vorticity of the fluid at the distribution plate. The SLM structure induced turbulent vortices in the surrounding region, and these vortices continuously altered the static pressure of the liquid. When the static pressure locally dropped below the vapor pressure of the liquid, cavitation occurred, leading to the formation of microbubbles [32,33]. In other words, additional microbubbles can be generated through vortex cavitation around the SLM structure on the distribution plate. Therefore, the proposed Venturi nozzle with an SLM structure enhanced microbubble generation through the combined effects of throat cavitation and vortex-induced cavitation at the distribution plate.

### 2.2. Experimental Setup for Selective Laser Melting

**Figure 2:**
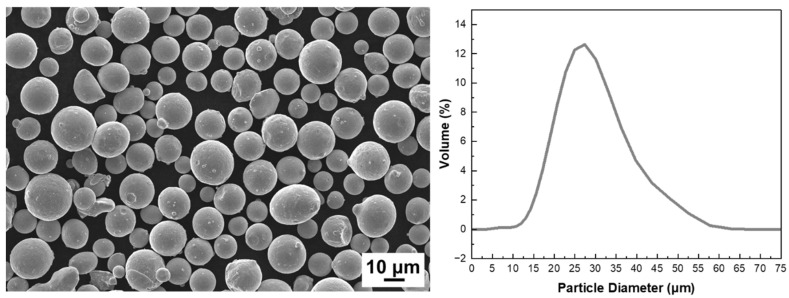
Powder morphology (**left**) and size distribution (**right**) of spherical SS316L powder.

Table 1 presents the chemical composition of the spherical SS316L (stainless steel 316L) powder used in this study. Figure 2 shows the morphology and particle size distribution of the SS316L powder used for the SLM process, with an average particle size of 19.35 μm.

**Figure 3:**
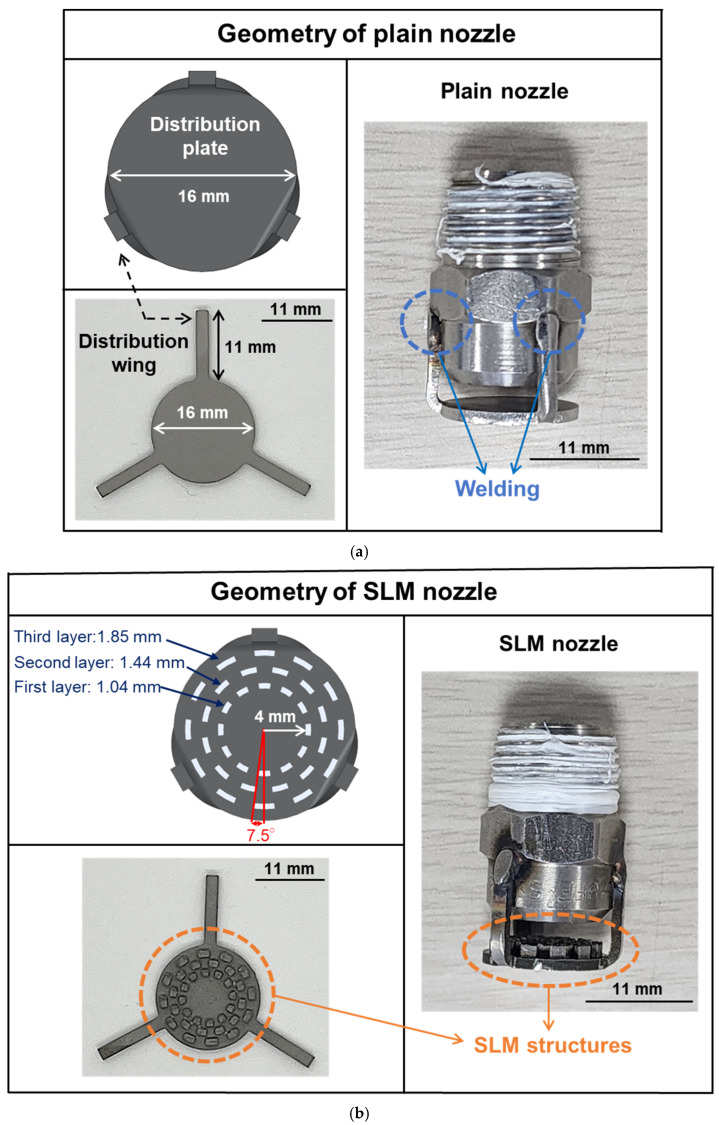
Schematic illustrations and images of the venturi-type nozzles with the distribution plates for the (**a**) Plain (i.e., distribution plate without the SLM structures) and (**b**) SLM nozzle (i.e., distribution plate with the SLM structures).

Figure 3 shows the SLM-fabricated structure produced on the distribution plate of the Venturi nozzle. The SLM process was employed for the fabrication. A 330 W continuous-wave fiber laser with a central wavelength of 1080 nm was used as the energy source. The laser beam had a beam size of 200 μm with a Gaussian energy distribution and a beam quality factor (*M*^2^) of 1.3. The laser beam was scanned over the SS316L powder bed using a biaxial galvanometer mirror scanner. The fabrication chamber, with dimensions of 250 (W) × 250 (L) × 100 (H) mm^3^, was purged with argon gas (purity: 99.99%), and the oxygen concentration inside the chamber was maintained below 0.5 vol% to prevent oxidation during the process. To optimize the SLM process parameters, six different conditions of SLM process were established by varying the laser power and scanning speed. The density and porosity of the fabricated SLM structures under each condition were evaluated, and the optimized processing condition was selected for the microbubble generation tests. The detailed SLM process parameters are listed in Table 2. The SLM structures with dimensions of 2 × 2 × 5 mm^3^, a layer thickness of 30 μm, and a hatch spacing of 120 μm were used for every conditions. The volumetric laser energy density was calculated using Equation (3), where E is the volumetric laser energy density (J/mm^3^), P is the laser power (W), H is the hatch distance (mm), V is the scan speed (mm/s), and T is the layer thickness (mm) [34].

(3) $$E=\frac{P}{H\cdot V\cdot T}$$

The laser scanning direction was rotated by 90° for subsequent layers to reduce anisotropy during fabrication. The SLM structure was built on a stainless steel 316L substrate, which was used as the distribution plate for the Venturi nozzle.

Figure 3a shows the schematic illustration and actual image of the Venturi nozzle without an SLM structure on the distribution plate, hereafter referred to as the plain nozzle. Figure 3b presents the schematic illustration and actual image of the Venturi nozzle incorporating the SLM structure on the distribution plate, hereafter referred to as the SLM nozzle. The diameter of the distribution plate was 16.6 mm, and the wings of the distribution plate were welded to the Venturi nozzle, ensuring precise alignment between the centers of the nozzle and the distribution plate. In the SLM nozzle, the sub-millimeter-sized SLM structures were fabricated directly on the distribution plate. Each SLM structure consisted of three layers, and the width and height of each structure were 1 mm, respectively. The first layer of the SLM structure was positioned 4 mm away from the center of the distribution plate. The lengths of the first, second, and third layers of the SLM structures were 1.04, 1.44, and 1.85 mm, respectively. In addition, the side walls expanded outward at an angle of 7.5° from the center of the structure. These SLM structures were designed to enhance vortex generation at the distribution plate and to investigate its effect on microbubble formation.

### 2.3. Density Measurement and Microstructure Characterization

The density of the SLM structures was measured using the Archimedes’ method [35]. Each sample was measured five times, and the average value was calculated. Prior to microstructural characterization, the SLM structures were ground using SiC abrasive papers (grit sizes: 200–2000). For a qualitative phase analysis, X-ray diffraction (XRD) measurements were performed for the SS316L powder and SLM structures. In addition, the porosity and microstructure of the SLM structures were examined using an optical microscope (OM; Eclipse LV150N, Nikon Corp, Tokyo, Japan) and a scanning electron microscope (SEM; EVO10, Carl Zeiss Microscopy GmbH, Oberkochen, Germany).

### 2.4. Microbubble Generation Test

**Figure 4:**
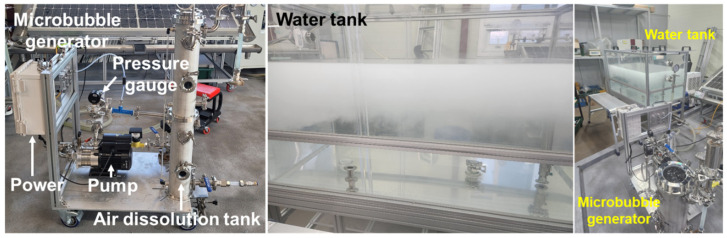
Pressurized dissolution-type microbubble generator (**left**), water tank (**center**), and combination of microbubble generator and water tank (**right**) for microbubble generation tests.

**Figure 5:**
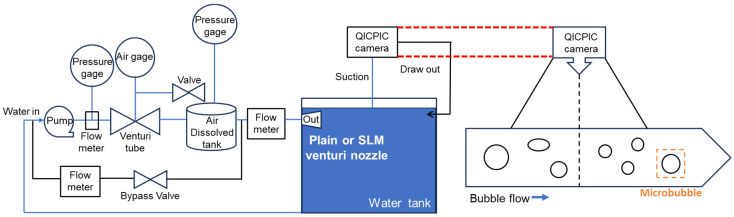
Schematic illustration of microbubble generator and dynamic image analysis method.

Figure 4 shows the experimental setup of the pressurized dissolution-type microbubble generation system used in this study. Water and air were used as the liquid and gas phases, respectively. Water was supplied from a 588 L tank (1200 (L) × 700 (W) × 700 (H) mm^3^) using a pump, and the flow conditions were controlled using a flow meter and a pressure gauge. Figure 5 illustrates a schematic diagram of the microbubble generation and observation system, along with the measurement principle of the dynamic image analysis method used to analyze microbubble formation.

Air was supplied through a Venturi-type tube, and the air flow rate was range from 100 to 700 cc/min. To dissolve air into water, a swirl-type mixer and an air dissolution tank maintaining a pressure of 0.2–0.3 MPa were employed. The fluid passing through the dissolution tank was divided into two streams through a control valve: one stream was directly discharged into the water tank, while another stream was bypassed back to the pump. This configuration allowed a portion of the pressurized dissolved water to be reused, thereby increasing the gas saturation level in the dissolution tank and promoting enhanced microbubble generation. By adjusting the bypass valve, both the bypass flow rate and the final pressure within the dissolution tank could be controlled. After being stored in the air dissolution tank, the air-saturated water was supplied back to the water tank through the Venturi nozzle at a flow rate of 30 L/min. Microbubbles were generated in the Venturi nozzle through the cavitation phenomenon. The number and size distribution of microbubbles were measured using a particle image analyzer (QICPIC, Sympatec, Clausthal-Zellerfeld, Germany) detection range from 0.55 µm to 33,792 µm with seven overlapping optical modules). The analyzer was connected to the water tank, and 150 mL of water was sampled for each measurement. The number and size of microbubbles were analyzed for 30 s, during which the measurement system captured images with dimensions of 2 (W) × 2 (L) × 0.2 (D) mm^3^ at a frame rate of 25 frames per second. During the experiments, the water temperature was maintained at 23 ± 1 °C. For the plain and SLM nozzles, the microbubble generation tests were repeated three times under seven different air flow rates (i.e., 100, 200, 300, 400, 500, 600, and 700 cc/min) and three different dissolution tank pressures (i.e., 0.2, 0.25, and 0.3 MPa). The averaged values obtained from the three repeated measurements were used for analysis.

### 2.5. Computational Fluid Dynamics Simulation

CFD simulations were conducted to elucidate the mechanism of microbubble generation in the SLM structure-assisted nozzle. ANSYS Fluent 2023 R2 (Ansys Inc., Canonsburg, PA, USA), a commercial CFD software package, was employed for the simulations. The analysis was based on the governing equations of mass conservation (Equation (4)) and momentum conservation (Equation (5)).

(4) $$\nabla \cdot \vec{V}=0$$

(5) $$\frac{\partial \vec{V}}{\partial t}+\vec{V}\cdot \nabla \vec{V}=-\frac{\nabla p}{\rho }+\frac{\mu }{\rho }\nabla^{2}\vec{V}+S_{M}$$

The *k*-*ε* turbulence model (Equation (6)) was employed to describe the flow behavior under turbulent conditions.

(6) $$\mu =\rho C_{\mu }\frac{k^{2}}{\epsilon }$$

Nomenclatures used in the numerical simulation are listed in Table 3. The CFD domain was limited to one-sixth of the distribution plate. The inlet condition was set to 30 L/min at 295 K, the bottom boundary was defined as a wall, and the top and rear boundaries were defined as pressure outlets (0 atm). Incompressible water was used as the working fluid, and 1000 iterations were performed under steady-state conditions.

## 3. Results and Discussion

### 3.1. Microstructure

To optimize the SLM process parameters, six different conditions of SLM process were evaluated. Density measurements were performed for each condition, and the optimal SLM process parameters were determined based on the density analysis results. The SLM process with a laser power of 140 W and a scanning speed of 100 mm/s was considered as the optimal laser parameters and used it to fabricate the SLM structures for the microbubble generation nozzle. Under this condition, the highest relative density of 97.76% was obtained, and a relatively low-density value may be obtained owing to the small build size of the SLM structures [36]. The detailed results are summarized in Table 2.

**Figure 6:**
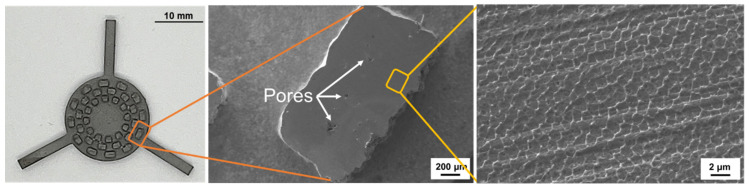
Low- and high-magnification SEM images of the SS316L structures, fabricated via the SLM process.

**Figure 7:**
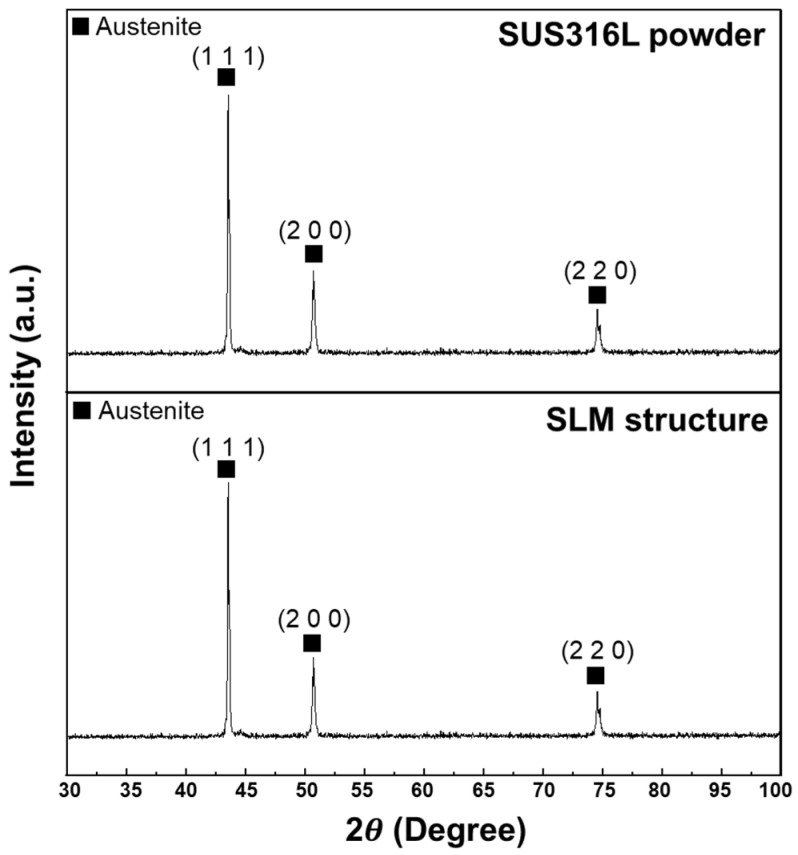
XRD pattern of SS316L for the powder and SLM structure.

Figure 6 shows the top-sectional SEM image of the SS316L SLM structure, which was fabricated by the optimal SLM process. Pores ranging from several micrometers to several tens of micrometers were observed within the microstructure of the SLM structure. Due to the presence of these pores, the density of the SLM-fabricated SS316L was measured to be 97.76% relative to conventional SS316L. The high-magnification SEM image within the yellow box reveals a sub-grain structure formed in the microstructure. This structure is attributed to rapid solidification and the Marangoni effect, which refers to fluid flow induced by surface tension gradients within the molten pool. These microstructural features are influenced by the temperature gradient and solidification rate within the melt pool during the SLM process [37,38,39,40]. Figure 7 presents the XRD analysis results of the SS316L powder and the SLM-fabricated SS316L specimen. No phase transformation was observed between the SS316L powder and SLM SS316L structure. All diffraction peaks corresponded to the austenite phase, indicating that the SLM process did not significantly affect the phase stability of SS316L [41].

### 3.2. Microbubble Generation Test

**Figure 8:**
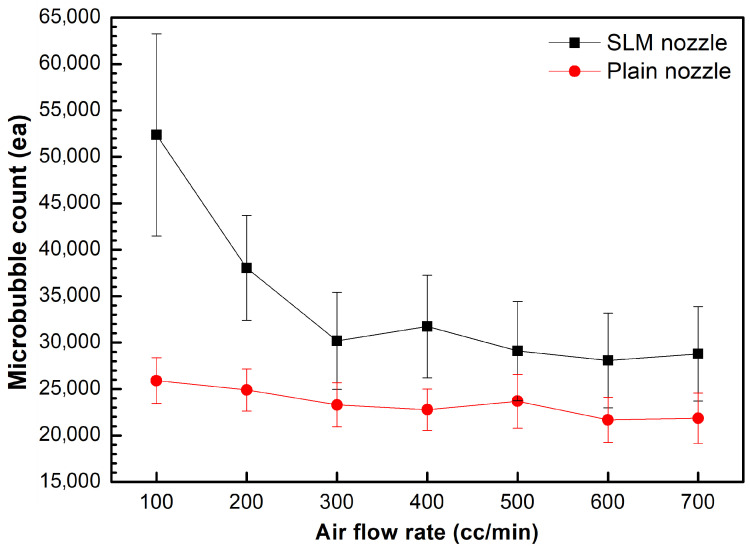
Number of generated microbubbles for the Plain and SLM nozzle at different air flow rates. The pressure of the pressurized dissolution tank was 0.3 MPa.

Air was introduced through a Venturi-type tube at flow rates ranging from 100 to 700 cc/min, and the number of generated microbubbles was measured at the pressure condition of the pressurized dissolution tank of 0.3 MPa, as shown in Figure 8. The number of generated microbubbles was strongly affected by the air flow rate. For both the Plain and SLM nozzles, the highest number of microbubbles was obtained at an air flow rate of 100 cc/min, whereas the number of generated microbubbles decreased with increasing air flow rate, owing to reduced air dissolution in the pressurized dissolution tank. The greater number of generated microbubbles was observed for the SLM nozzle compared with that of the Plain nozzle at every air flow rate. Accordingly, further microbubble generation tests were carried out at an air flow rate of 100 cc/min.

**Figure 9:**
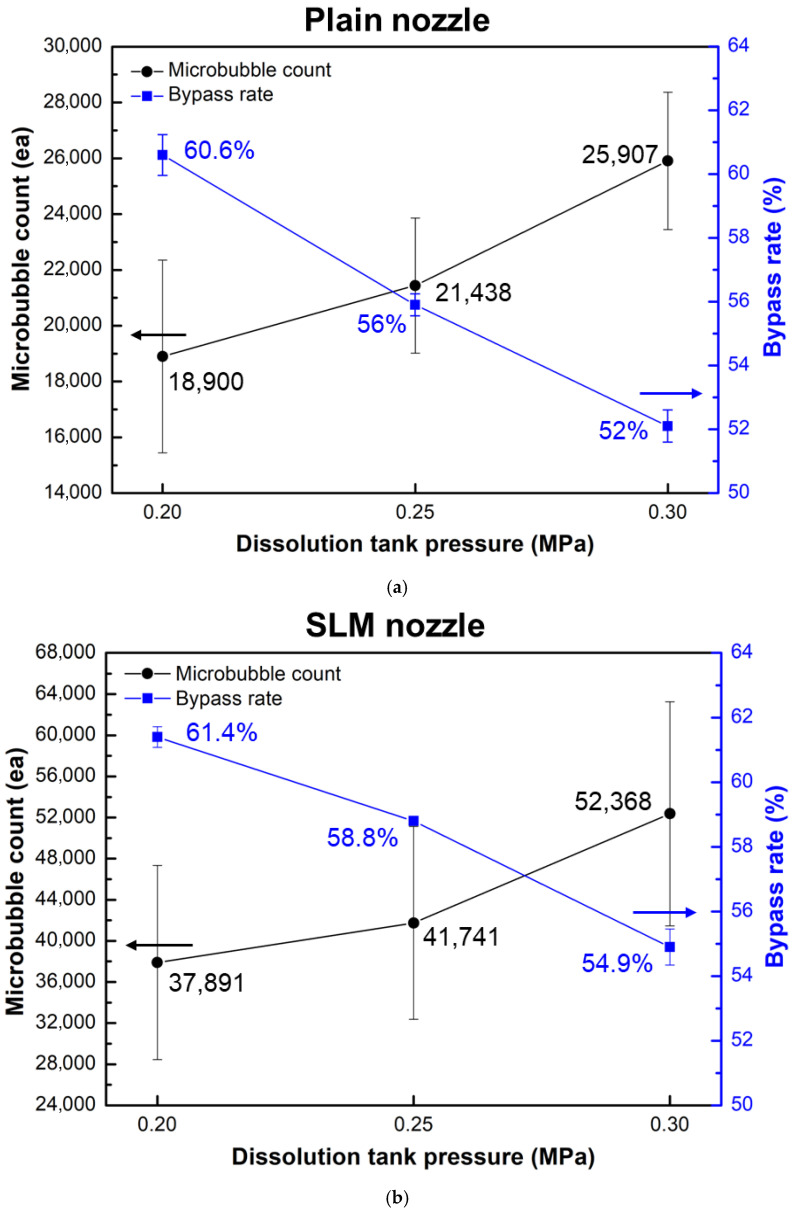
Number of generated microbubbles for the (**a**) Plain and (**b**) SLM nozzle at different dissolution tank pressures.

In addition, three pressure conditions (i.e., 0.2, 0.25, and 0.3 MPa) of the pressurized dissolution tank were applied to compare the bubble generation performance under an air flow rate of 100 cc/min and a water flow rate of 30 L/min. Figure 9a,b show the number of microbubbles generated by each nozzle type. For the plain nozzle, the average value of generated microbubbles was 18,900 under a pressure condition of 0.2 MPa. As the pressure of the pressurized dissolution tank increased, the number of generated microbubbles showed an increasing trend, reaching 25,907 at 0.3 MPa. For the SLM nozzle, a higher number of microbubbles was generated at the same pressure condition compared with the plain nozzle. The average values of generated microbubbles were 37,891, and 52,368 under a pressure condition of 0.2 and 0.3 MPa, respectively. The generated microbubble via the SLM nozzle at 30 MPa was increased by 102.1% compared with that of the Plain nozzle. In addition, the variation range of generated microbubble from repeated tests was larger for the SLM nozzle than that of the plain nozzle. This may be attributed to a large variation in fluid velocity associated with an increase in turbulent flow (verified in Section 3.3). The bypass ratio was designed to decrease as the pressure of the pressurized dissolution tank increased. For the plain nozzle, when the pressure increased from 0.2 MPa to 0.3 MPa, the bypass ratio decreased by 8.4%. In the case of the SLM nozzle, the bypass ratio decreased by 6.5% under the same pressure variation. Even under identical pressure conditions, the bypass ratios of the pressurized dissolution tank differed by approximately 2% between the plain nozzle and the SLM nozzle. This difference was attributed to variations in flow resistance within the pump–tank–bypass circuit, the dynamic adjustment characteristics of the control valve, and minor changes in fluid properties, which collectively influence the flow distribution within the system.

**Figure 10:**
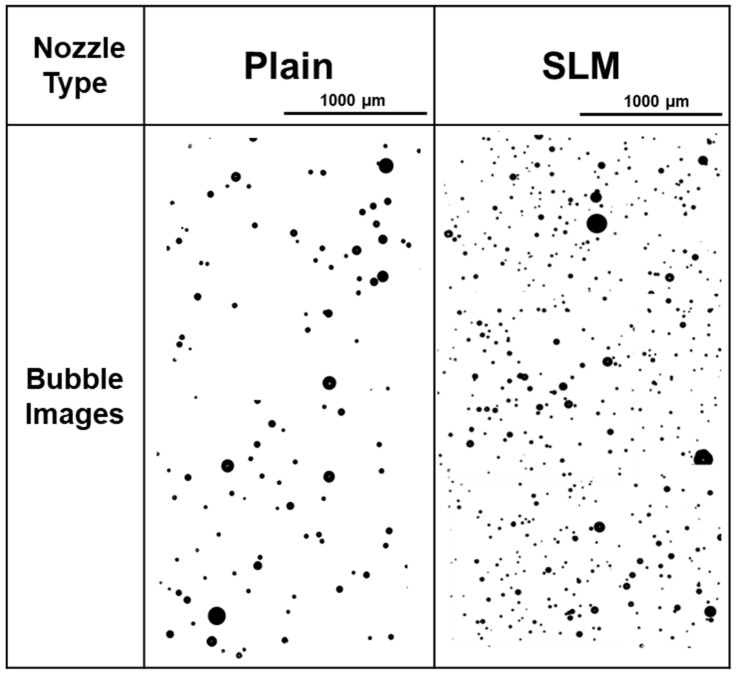
Generated microbubble image from the particle image analyzer for the Plain and SLM nozzle at a dissolution tank pressure of 0.3 MPa.

Figure 10 shows the images of generated microbubble at a dissolution tank pressure of 0.3 MPa via the particle image analyzer, with an image size of 2 (W) × 3.8 (L) × 0.2 (D) mm^3^. A denser microbubble distribution was observed for the SLM nozzle compared with the plain nozzle. The sizes of the generated microbubbles from the plain and SLM nozzles were distributed in the range of 1–110 μm, which satisfied the general size condition of microbubbles [10]. For the plain nozzle, the average microbubble diameter was 38.8 μm, and the highest frequency of microbubbles was observed in the size range of 31–40 μm. In contrast, the SLM nozzle exhibited a different microbubble size distribution. The largest number of microbubbles was observed in the size range of 21–30 μm, and the average microbubble diameter was 31.82 μm, which was significantly smaller than general microbubble size generated by the typical Venturi-type nozzles (approximately a few hundred micrometers) [23,24,25].

### 3.3. Computational Fluid Dynamics Simulation

**Figure 11:**
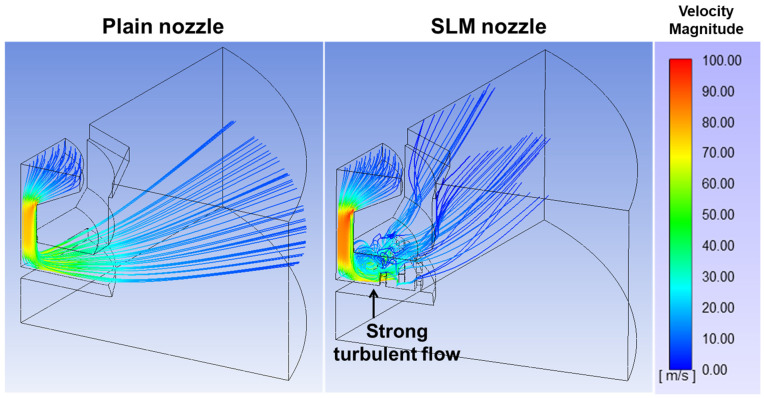
Velocity magnitude and streamline from computational fluid dynamics simulation for the Plain and SLM nozzles.

To investigate the microbubble generation mechanism of each nozzle, CFD simulations were conducted using ANSYS Fluent, with particular emphasis on fluid velocity and streamline over the distribution plate. Vortex cavitation refers to cavitation occurring within turbulent flows (i.e., vortex) in the fluid. Such vortical structures generate localized low-pressure zones, leading to liquid vaporization and consequently facilitating microbubble generation. As mentioned earlier, the SLM structures were fabricated on the distribution plate to induce turbulent vortices, thereby promoting microbubble generation through vortex-induced cavitation. Figure 11 shows the fluid velocity magnitude and streamline at the distribution plate for the Plain and SLM nozzles obtained via CFD simulation. Compared with the Plain nozzle, a strong turbulent flow was generated at the SLM structure for the SLM nozzle. This behavior was attributed to the presence of the SLM structures, which induced irregular turbulent flow patterns. These results indicated that the SLM-assisted nozzle enhanced cavitation effects due to the SLM structures, which induced rapid variations in fluid velocity and pressure as well as increased turbulent flow. As a result, bubbles were fragmented into smaller sizes, leading to the generation of a larger number of microbubbles compared with the plain nozzle [10]. Therefore, the introduction of SLM structures promoted turbulence-induced cavitation and bubble fragmentation, resulting in a higher number of smaller microbubbles.

## 4. Conclusions

In this study, the SLM process was applied to fabricate surface structures on the distribution plate of Venturi-type nozzle used in a pressurized dissolution-type microbubble generator. The number and size distribution of generated microbubbles from both the plain nozzle and the SLM nozzle were investigated. The main findings of this study can be summarized as follows:

(1) Fabrication of the SLM-structured Venturi nozzle: The novel design of Venturi nozzle was fabricated with the sub-millimeter-sized SLM-assisted structures, which had difficulty to fabricate using the conventional milling or other machining methods. The SLM structures were formed on the distribution plate of Venturi nozzle under the optimal condition of SLM process, as follows: a layer thickness of 30 μm, a hatch spacing of 120 μm, a laser power of 140 W, and a scanning speed of 100 mm/s.
(2) Microbubble generation tests: Microbubble generation tests were conducted for 30 s under different air flow rates and dissolution tank pressures. The largest microbubble was generated at an air flow rate of 100 cc/min and a dissolution tank pressure of 0.3 MPa. The number and size distribution of the generated microbubbles were measured using a particle image analyzer. For both the plain and SLM nozzles, the generated microbubble sizes ranged from 1 to 110 μm, satisfying the general size condition of microbubbles. In the plain nozzle, the largest number of generated microbubbles was 25,907, and the average bubble diameter was 38.8 μm. As the pressure of the pressurized dissolution tank increased, the number of generated microbubbles also increased. For the SLM nozzle, the number of microbubbles increased while the bubble size decreased, compared with the Plain nozzle. The greatest number of generated microbubbles was 52,368, with an average bubble diameter of 31.82 μm.
(3) Effect of SLM structures on the microbubble generation: The experimental and CFD simulation results demonstrated that the SLM structures on the distribution plate enhanced the turbulence intensity of the fluid flow and induced rapid pressure fluctuations, which promoted the cavitation phenomenon. As a result, bubble fragmentation was enhanced, leading to an increased number of microbubbles. These findings confirmed that the SLM-fabricated structure played a significant role in improving microbubble generation characteristics. Therefore, the proposed SLM-structured Venturi nozzle provided an effective approach for enhancing microbubble generation performance in pressurized dissolution systems.

## Figures and Tables

**Table 1:** Chemical composition of the spherical SS316L powder.

Elements	C	Cr	Cu	Mn	Mo	Ni	P	S	Si	Fe
wt%	0.03	17.5	0.50	2.0	2.25	12.5	0.025	0.01	0.75	Bal

**Table 2:** Experimental conditions and measured density for the SLM SS316 specimens.

Specimens	Laser Power (W)	Scan Speed (mm/s)	Laser Energy Density (J/mm^3^)	Density (%)
SLM (100/100)	100	100	278	96.93
SLM (100/1000)	100	1000	28	95.55
SLM (140/100)	140	100	389	97.76
SLM (140/1000)	140	1000	39	96.78
SLM (180/100)	180	100	500	95.94
SLM (180/1000)	180	1000	50	93.79

**Table 3:** Nomenclatures used in CFD simulations.

Symbol	Nomenclature	Symbol	Nomenclature
P	Static pressure of liquid	ρ	Density of liquid
ε	Rate of dissipation of turbulent kinetic energy	*S_M_*	Source term
*V*	Liquid velocity	$C_{\mu }$	Adjustable constants, 0.09
μ	Viscosity	K	Turbulent kinetic energy

## Data Availability

The data presented in this study are available on request from the corresponding author.
